# Perceived message effectiveness and readiness to quit among youth in Guangdong: The mediating role of self-efficacy and the moderating effect of nicotine dependence

**DOI:** 10.1097/MD.0000000000048756

**Published:** 2026-05-08

**Authors:** Yang Zhao, Xuelan Wu, Xiaoyu Tao

**Affiliations:** a Faculty of Humanities and Arts, Macau University of Science and Technology, Taipa, Macao SAR, China; b Faculty of Medicine, Macau University of Science and Technology, Taipa, Macao SAR, China; c School of Management, Guangzhou College of Commerce, Guangzhou, Guangdong, China; d Department of Lifestyle Medicine, Hong Kong Public Health Technology Research Center, Kowloon, Hong Kong SAR, China.

**Keywords:** nicotine dependence, perceived message effectiveness, self-efficacy

## Abstract

Tobacco use among adolescents in China poses a serious public health challenge, with Guangdong being a key region for intervention. Understanding the psychological mechanisms of perceived message effectiveness (PME) can inform more effective tobacco control strategies. This study aimed to examine how PME influences quitting readiness among youth smokers in Guangdong, with self-efficacy as a mediator and nicotine dependence as a moderator. A cross-sectional survey was conducted among youth smokers recruited from schools and community centers in Guangdong between August and September 2025, using self-administered questionnaires. PME was assessed using a standardized short scale that evaluates the perceived persuasiveness of anti-smoking messages. Participants aged 15 to 24 years were selected using stratified cluster sampling from secondary schools, vocational colleges, and community centers across Guangdong province. Participants completed validated measures, including the Hooked on Nicotine Checklist, the Smoking Self-Efficacy Questionnaire-12, and the Readiness/Contemplation Ladder. Correlation, mediation, moderation, and moderated mediation analyses were performed using SPSS version 26.0 and the PROCESS macro with bootstrapping. PME was positively correlated with both self-efficacy (*r* = 0.42, *P* < .001) and readiness to quit (*r* = 0.36, *P* < .001), indicating moderate associations consistent with behavioral research expectations. Mediation analyses indicated that self-efficacy significantly mediated the PME-readiness relationship (indirect standardized effect *β* = 0.20, 95% confidence interval [0.11, 0.30]), accounting for approximately 55% of the total effect. Nicotine dependence moderated both the PME-self-efficacy (interaction *β* = –0.12, *P* = .015) and the self-efficacy-readiness pathways (interaction *β* = –0.14, *P* = .018). The indirect effect of PME on readiness was significant among low-dependence youth but not among highly dependent youth. PME promotes quitting readiness mainly through self-efficacy, but this pathway is weakened among youth with higher nicotine dependence. Tailored health communication strategies should therefore combine efficacy-building messages with additional support for highly dependent smokers. The findings suggest that public health campaigns aiming to increase quitting readiness among youth should focus on improving the perceived persuasiveness of anti-smoking messages and strengthening self-efficacy, particularly by providing extra support for those with higher nicotine dependence.

## 1. Introduction

Tobacco use among adolescents and young adults continues to pose a formidable threat to public health globally, and China remains a pivotal battleground in this struggle. A meta-analysis covering 131 studies in China estimated that the pooled prevalence of smoking among youth is 8.17%.^[1]^ In Shenzhen, a school-based cross-sectional study reported a current smoking prevalence of 2.3% among adolescents (male: 3.4%; female: 1.0%).^[2]^ These statistics underscore the importance of focusing tobacco control efforts on youth, especially in economically dynamic provinces such as Guangdong. Since 2015, Guangdong province has implemented multiple tobacco control initiatives, including mass media campaigns, school-based health education, and community outreach activities emphasizing the harms of smoking and the benefits of cessation. These campaigns are periodically renewed and have been integrated into broader provincial health promotion programs targeting adolescents. Although multiple national and provincial anti-smoking campaigns have been implemented since 2015, systematic evaluations of their effectiveness remain scarce, especially among adolescent and young adult populations. This lack of evidence underscores the need to assess perceived message effectiveness (PME) as an indicator of communication impact in youth-targeted tobacco control.

Persuasive health communication forms one of the cornerstones of tobacco control, with anti-smoking campaigns frequently relying on message framing, emotional appeal, and perceived credibility to influence risk perceptions, attitudes, and behavioral intentions. PME refers to an individual’s judgment of how persuasive and convincing a health message is in influencing attitudes or behaviors. Within the behavioral change process, PME acts as a proximal cognitive response linking message exposure to key mediators such as self-efficacy and, ultimately, to behavioral intentions. Among the tools used in message evaluation, PME stands out: meta-analytic evidence suggests PME is modestly predictive of readiness to quit and cessation behavior.^[3]^ PME has been widely used in pretesting campaigns to approximate likely impact among target audiences. However, PME is a proximal indicator; its predictive validity is likely mediated by the recipients’ psychological responses, among which self-efficacy is a central candidate.

Self-efficacy, or confidence in one’s ability to execute behaviors necessary to manage prospective scenarios, is fundamental in social cognitive and health behavior theories. In smoking cessation research, self-efficacy to resist cravings, cope with withdrawal, and reject cigarettes has robustly predicted both intention and successful quitting.^[4]^ Daily within-person fluctuations in self-efficacy also predict moment-to-moment resistance to smoking in ecological momentary assessment studies.^[5]^ In message impact frameworks, persuasive messages may heighten recipients’ perceived agency, which then translates to stronger behavioral intention: a mediation logic with empirical support in domains such as nutrition messaging and physical activity promotion. Thus, in the anti-smoking context, a plausible pathway is: PME → self-efficacy → quitting intention.

**Figure 1.**
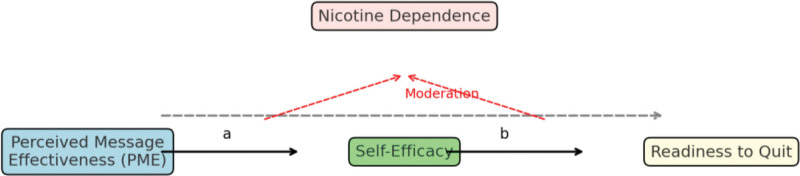
Conceptual framework of perceived message effectiveness and quitting readiness. The hypothesized model illustrates the direct effect of PME on readiness to quit smoking, the indirect effect mediated by smoking self-efficacy, and the moderating role of nicotine dependence on the PME-self-efficacy and self-efficacy-readiness pathways. Covariates (age, sex, education level, and smoking frequency) were controlled for in all analyses. PME = perceived message effectiveness.

Nonetheless, the efficacy of that mediation pathway may not be uniform across all smokers. High nicotine dependence can impose physiological constraints (e.g. stronger cravings, withdrawal, lower perceived control) that attenuate responsiveness to persuasive messages or hinder the translation of confidence into intention or behavior. Indeed, empirical work in China has found that psychological determinants of quitting intention differ across dependence strata. For example, in a Chinese sample applying Protection Motivation Theory, dependence moderated relationships among cognitive appraisal constructs and quit intention.^[4]^ Moreover, a study of Chinese male smokers, Huang et al (2019) found that cigarette dependence moderated how smoking rationalization beliefs related to motivational strength, suggesting that dependence can reshape the receptivity to cognitive or persuasive influences.^[6]^ Hence, nicotine dependence might moderate both the effect of PME on self-efficacy (i.e. how much message persuasiveness can boost perceived agency) and the effect of self-efficacy on quitting intention (i.e. whether confidence effectively leads to intention), forming a moderated mediation model. The hypothesized relationships among PME, self-efficacy, nicotine dependence, and readiness to quit are summarized in Figure 1.

Despite the implementation of the National Tobacco Control Plan and various provincial anti-smoking measures, youth smoking and nicotine dependence remain persistent challenges in China. Surveys in China have reported persistent nicotine dependence among youth smokers.^[1,2]^ However, limited research has examined how persuasive anti-smoking messages translate into psychological readiness to quit among this vulnerable group. Addressing this gap can help optimize youth-targeted communication strategies within China’s evolving tobacco control framework. Within China, relatively few studies have examined how persuasive anti-smoking messages operate among youth smokers, particularly in Guangdong.

To this end, the present study aims to investigate whether PME is associated with quitting readiness, whether this relationship is mediated by self-efficacy, and whether nicotine dependence moderates the direct or indirect pathways. By integrating validated instruments such as the Hooked on Nicotine Checklist (HONC), Smoking Self-Efficacy Questionnaire-12 (SEQ-12), and the Readiness/Contemplation Ladder, this study seeks to provide robust empirical evidence on the psychological processes through which anti-smoking messages may influence readiness to quit among Guangdong youth.

Based on the conceptual framework presented in Figure 1, the following null hypotheses were tested:

H0_1_: There is no association between PME and readiness to quit;

H0_2_: Self-efficacy does not mediate the relationship between PME and readiness to quit;

H0_3_: Nicotine dependence does not moderate the relationship between PME and self-efficacy, nor the relationship between self-efficacy and readiness to quit.

## 2. Methods

### 2.1. Study design and participants

The research used a cross-sectional survey design and psychological and behavioral correlates intention toward smoking cessation among adolescents in Guangdong province, China. Based on its ability to estimate prevalence, pinpoint correlations, and develop hypotheses on the possible mechanisms at play within a population at a single time, a cross-sectional method is the most appropriate.^[7]^ The target population for the study is youth smokers, who are in a developmental stage, which makes them particularly vulnerable to becoming dependent on nicotine and to the use of compelling communication aimed at changing health behavior. Participants were required to have smoked at least 1 cigarette in the past 30 days, which is inline with current smoking for epidemiological definitions.^[7]^

To achieve a cross-sectional representation of youth from diverse backgrounds in Guangdong, a stratified cluster sampling approach was adopted to ensure coverage of both urban and peri-urban areas. Five secondary schools, six vocational colleges, and 3 community centers were randomly selected from the provincial education and health department registries. Within each selected cluster, classes or activity groups were randomly chosen to participate. Eligible participants were youth aged 15 to 24 years who had smoked at least 1 cigarette in the past 30 days. Of 520 invited individuals, 482 provided valid responses (response rate = 92.7%). All respondents provided written informed consent, and for minors under 18 years, parental consent was additionally obtained. This study focused exclusively on traditional combustible cigarette smoking. All survey items referred specifically to cigarette use. Participants who reported using only e-cigarettes or other noncombustible tobacco products were excluded from the final analysis.

This study did not involve experimental randomization; instead, random selection was applied solely during sampling to enhance representativeness. This sampling strategy maximized external validity and minimized selection bias.

To ensure adequate precision for the planned moderated mediation analyses with bootstrapped CIs (confidence intervals), an a priori sample size evaluation was conducted using G*Power 3.1. Assuming a small-to-moderate effect size (*f*^2^ = 0.05), *α* = 0.05, power = 0.90, and up to 8 predictors in multiple regression models, the minimum required sample under simple random sampling was approximately 300. Considering the use of stratified cluster sampling, a design effect of 1.5 was applied to account for clustering, yielding a required sample of about 450. We therefore targeted ≥ 450 participants and ultimately achieved 482, which provided sufficient statistical power for all planned analyses.

Before the formal survey, a pilot test was conducted among 30 youth smokers from 1 vocational college and 1 community center in Guangzhou to assess the clarity and comprehension of all questionnaire items. Minor wording adjustments were made based on participants’ feedback to ensure cultural appropriateness and clarity. Data from the pilot participants were excluded from the main analyses.

### 2.2. Inclusion and exclusion criteria

Participants were eligible for inclusion if they met the following criteria:

1. aged between 15 and 24 years;
2. had smoked at least 1 cigarette in the past 30 days;
3. were recruited from selected schools or community centers in Guangdong province; and
4. provided informed consent (with parental consent obtained for participants under 18 years of age).

Participants were excluded if they:

1. reported exclusive use of e-cigarettes or other noncombustible tobacco products;
2. provided incomplete or inconsistent questionnaire responses; or
3. declined or were unable to provide informed consent.

### 2.3. Measures

#### 2.3.1. Sociodemographic characteristics

Respondents provided their age, sex, highest level of education, and some information about their family. These demographic characteristics were included in the study both for purposes of description and for use as potential covariates in the regression analysis.

#### 2.3.2. Nicotine dependence

Nicotine dependence was assessed using the HONC, which contains 10 items evaluating loss of autonomy over tobacco use.^[8]^ Each “yes” response is scored 1, producing a total from 0 to 10, with higher scores indicating stronger dependence. Participants scoring ≥ 1 were classified as having nicotine dependence, consistent with prior epidemiological studies.

Although the HONC was originally designed for early adolescents, it has been validated and widely applied among older youth and young adults. The Chinese version demonstrated good reliability (Cronbach α ≈ 0.83) and validity among Chinese-speaking adolescent smokers.^[9]^ This supports its applicability to our 15 to 24-year-old sample.

Both continuous scores and categorical classifications were used in analyses to reflect variations in dependence severity and to test moderation effects in the mediation models.

#### 2.3.3. Self-efficacy

SEQ-12 was used to examine the level of confidence the participants had in their ability to control smoking in internal (e.g., stress, negative affect) and external (e.g., in social activities, offers from peers) situations. The SEQ-12 has subscale and total mean scores whereby higher scores depict higher levels of self-efficacy. This instrument has been used in various cultures and found to be a useful predictor of cessation outcomes.^[10]^ The internal consistency of the SEQ-12 has been previously validated in Chinese populations, with Cronbach alpha values reported to be above 0.80.

#### 2.3.4. Readiness to quit

Readiness to quit was measured using the Readiness/Contemplation Ladder, a validated 11-point visual analogue scale ranging from 0 (“no thought of quitting”) to 10 (“taking action to quit”). Higher scores reflect greater quitting readiness. The scale has shown good validity in predicting cessation intention and behavior in both Western and Chinese samples.^[11,12]^

#### 2.3.5. PME

The effectiveness of the anti-smoking campaign was evaluated using a short standardized scale that measures the PME of participants. PME metrics function as proximal measures of a message’s capacity to shift perspective and have been shown to correlate with intention and outcome behaviors, as well as with attitude and behavior change.^[3]^ The internal consistency of the PME scale has also been supported in previous studies conducted in Chinese populations, with reported Cronbach alpha values exceeding 0.80.

### 2.4. Procedures

Data collection was done between August and September 2025. Trained research assistants under ethical training concerning compliance and confidentiality along with administration of the surveys conducted them in both classrooms and community settings. Participants completed the paper-based questionnaires individually to reduce peer influence and social desirability bias. Anonymity was emphasized throughout the process, and no personally identifying information was recorded. All completed questionnaires were checked for completeness on-site, sealed in envelopes, and securely transferred for data entry.

### 2.5. Statistical analysis

All analyses were performed using SPSS version 26.0 (IBM Corp., Armonk) and R version 4.2. Descriptive statistics were used to summarize demographic characteristics and scale distributions. Group differences were examined using *t*-tests or analysis of variance (ANOVA) for continuous variables and chi-square tests for categorical variables. Pearson correlation coefficients were calculated to assess associations among PME, self-efficacy (SEQ-12), nicotine dependence (HONC), and readiness to quit scores.

Prior to inferential analyses, all continuous variables were tested for normality using skewness, kurtosis, and visual inspection of histograms and *Q*–*Q* plots. The distributions of PME, self-efficacy, readiness to quit, and nicotine dependence were within acceptable ranges (absolute skewness < 2, kurtosis < 7), supporting the use of parametric tests.

The hypothesized moderated mediation model (Fig. 1) examined whether PME influenced readiness to quit both directly and indirectly via self-efficacy, and whether nicotine dependence moderated the indirect pathway. Analyses were conducted using the PROCESS macro (Model 14)^[13]^ with 5000 bootstrap resamples and bias-corrected 95% CIs. An indirect effect (*β* < sub > indirect </sub >) was defined as the product of the path coefficients for PME → self-efficacy (a-path) and self-efficacy → readiness (b-path), and was considered significant when the 95% CI did not include zero. The index of moderated mediation quantified whether this indirect effect differed across dependence levels.

In all models, age, gender, education level, and daily cigarette consumption were included as covariates to control for potential confounding, consistent with prior tobacco control research highlighting their influence on smoking behavior and cessation readiness.^[12]^ Statistical significance was set at *P* < .05 (2-tailed).

### 2.6. Ethical considerations

The study protocol was reviewed and approved by the Ethics Committee of Hong Kong Public Health Technology Research Center (Ref. No.: PHTR/25/009-0003). All study procedures adhered to the ethical principles of the Declaration of Helsinki.^[14]^ Participation was strictly voluntary, and participants could withdraw at any stage without penalty. Confidentiality was ensured through anonymized data collection and secure storage. For underage participants, both parental consent and adolescent assent were required prior to participation.

## 3. Results

This section presents the findings of the study, which aimed to examine the association between PME and readiness to quit, as well as the mediating role of self-efficacy and the moderating effect of nicotine dependence. A total of 482 youth smokers from Guangdong province were included in the final analysis. The mean age of participants was 19.3 years (standard deviation [SD] = 2.4), and 61.6% were male. Most participants were enrolled in vocational colleges (43.2%), followed by universities (31.1%) and secondary schools (25.7%). On average, participants smoked on 12.5 days (SD = 8.7) in the past month, with a mean daily cigarette consumption of 6.2 (SD = 4.5). According to the HONC, 58.7% of the sample met the criteria for nicotine dependence.

Scale scores demonstrated moderate PME perceptions (M = 3.53, SD = 0.45), mid-range self-efficacy (SEQ-12: M = 3.06, SD = 0.48), and average readiness to quit of 5.96 (SD = 1.88). Nicotine dependence scores averaged 3.97 (SD = 2.14).

### 3.1. Correlational analysis

**Figure 2.**
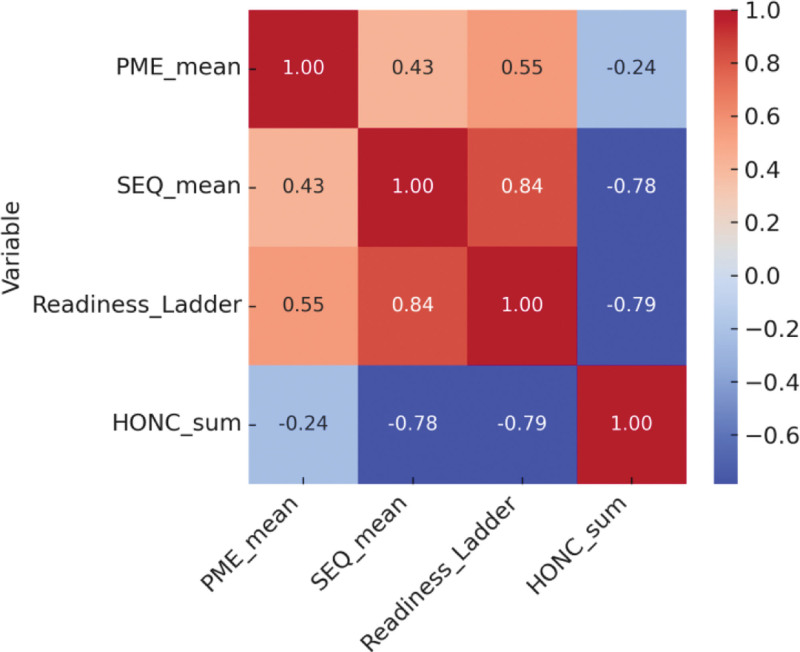
Correlation matrix of perceived message effectiveness, self-efficacy, readiness to quit, and nicotine dependence. The heatmap displays Pearson correlation coefficients among PME, smoking self-efficacy (SEQ-12), readiness to quit, and nicotine dependence (HONC). Warmer colors indicate stronger positive correlations, while cooler colors indicate negative correlations. All correlations shown are statistically significant (*P* < .001). HONC = Hooked on Nicotine Checklist, PME = perceived message effectiveness, SEQ-12 = Smoking Self-Efficacy Questionnaire-12.

As illustrated in Figure 2, PME was positively correlated with self-efficacy (*r* = 0.42, *P* < .001) and readiness to quit (*r* = 0.36, *P* < .001). Self-efficacy also correlated strongly with readiness (*r* = 0.48, *P* < .001). Nicotine dependence showed significant negative correlations with both self-efficacy (*r* = –0.31, *P* < .001) and readiness (*r* = –0.28, *P* < .001).

### 3.2. Mediation analysis

PROCESS mediation analysis revealed that self-efficacy significantly mediated the relationship between PME and readiness (see Table 1). The indirect effect of PME on readiness through self-efficacy was significant (*β* = 0.20, standard error (SE) = 0.05, 95% CI [0.11, 0.30]). The direct effect of PME on readiness remained significant (*β* = 0.16, SE = 0.07, *P* = .021), indicating partial mediation. Approximately 55% of the total effect of PME on readiness was explained by the indirect pathway via self-efficacy.

**Table 1:** Mediation path coefficients.

Path	Model	Interpretation	Coef	SE	*t*	*P*	*R* ^2^
a-path	SEQ ~ PME	PME → SEQ	0.47	0.04	11.00	< .01	0.19
b-path	Readiness ~ SEQ + PME	SEQ → Readiness	2.87	0.09	30.57	< .01	0.75
c’ path	Readiness ~ SEQ + PME	PME → Readiness (direct)	0.99	0.10	9.75	< .01	0.75
c path (total)	Readiness ~ PME	PME → Readiness (total)	2.33	0.15	15.29	< .01	0.31
indirect (a*b)	Derived	PME → SEQ → Readiness	1.34				

This table presents the results of the mediation analysis examining the indirect effect of PME on readiness to quit through self-efficacy. The indirect effect (*β*_indirect) represents the product of the PME → self-efficacy (a-path) and self-efficacy → readiness (b-path). A 95% CI that does not include zero indicates a statistically significant mediation effect. CI = confidence interval, PME = perceived message effectiveness, SE = standard error, SEQ = Smoking Self-Efficacy Questionnaire-12.

### 3.3. Moderation analysis

Nicotine dependence significantly moderated the relationship between PME and self-efficacy for quitting. The interaction term PME × HONC was significant (*β* = –0.12, SE = 0.05, *P* = .015), indicating that higher nicotine dependence weakened the positive association between PME and self-efficacy.

**Figure 3.**
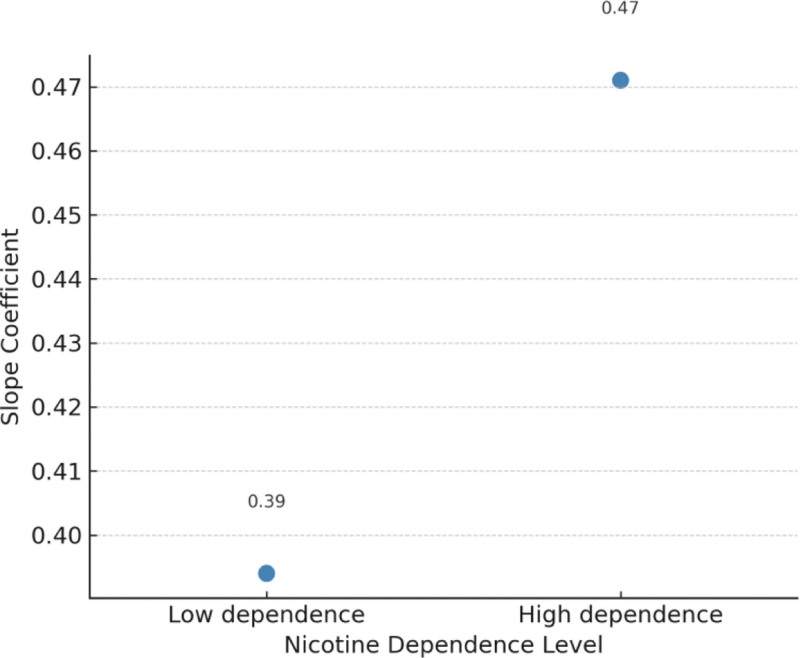
Moderating effect of nicotine dependence on the association between perceived message effectiveness and self-efficacy. Simple slopes analysis illustrates the relationship between PME and smoking self-efficacy (SEQ-12) at low (−1 SD) and high (+1 SD) levels of nicotine dependence (HONC). The slope is steeper for participants with lower nicotine dependence, indicating a stronger positive association, while the slope is attenuated among those with higher dependence. HONC = Hooked on Nicotine Checklist, PME = perceived message effectiveness, SD = standard deviation, SEQ-12 = Smoking Self-Efficacy Questionnaire-12.

Simple slopes analysis (Fig. 3) further demonstrated that PME predicted self-efficacy more strongly among participants with low dependence (*β* = 0.41, *P* < .001) compared with those with high dependence (*β* = 0.18, *P* = .071). As illustrated in Figure 3, the slope for low-dependence participants was noticeably steeper, confirming that nicotine dependence attenuated the motivational influence of message effectiveness on self-efficacy.

Similarly, nicotine dependence moderated the path from self-efficacy to readiness to quit (*β* for interaction = –0.14, SE = 0.06, *P* = .018). This association was robust among low-dependence youth (*β* = 0.52, *P* < .001) but substantially weaker and nonsignificant among highly dependent youth (*β* = 0.19, not significant).

### 3.4. Moderated mediation analysis

The moderated mediation model confirmed that the indirect effect of PME on readiness to quit through self-efficacy varied by the level of nicotine dependence (see Table 2). Among participants with low nicotine dependence, the indirect effect was significant (*β* = 0.24, 95% CI [0.14, 0.36]), whereas among those with high dependence, the effect was attenuated and not significant (*β* = 0.08, 95% CI [–0.01, 0.19]). The index of moderated mediation was statistically significant (index of moderated mediation = –0.09, 95% CI [–0.18,–0.02]), indicating that higher dependence levels weakened the indirect influence of PME on readiness via self-efficacy.

**Table 2:** Moderated mediation effects summary table.

Quantity	Point_Estimate	Boot_CI_Lower (95%)	Boot_CI_Upper (95%)	Boot_N
i.e.(W = 0 nondependent tobacco)	0.93	0.57	1.36	1000
i.e.(W = 1 Tobacco-dependent)	1.34	1.09	1.60	1000
IMM = i.e.(1) - i.e.(0)	0.40	-0.08	0.86	1000

This table summarizes the moderated mediation analysis, including the interaction effects and conditional indirect effects at different levels of nicotine dependence. The IMM indicates whether the indirect effect of PME on readiness through self-efficacy varies across levels of nicotine dependence. A 95% CI that does not include zero indicates a statistically significant moderated mediation effect.

“W” represents nicotine dependence (the moderator), coded as 0 = nondependent tobacco and 1 = tobacco-dependent.

CI = confidence interval, IMM = index of moderated mediation, PME = perceived message effectiveness.

Table 2 summarizes the standardized regression coefficients (*β*), SE, and 95% CIs for all paths in the mediation and moderated mediation analyses. The table corresponds to the conceptual model depicted in Figure 3 and reports the direct, indirect, and interaction effects among PME, self-efficacy, readiness to quit, and nicotine dependence.

### 3.5. Additional analyses

Multivariable regression models controlling for demographic covariates (age, sex, and education) and smoking behaviors (frequency and daily consumption) yielded consistent results. All primary effects and interactions remained significant at comparable magnitudes. Variance inflation factors (all < 2.1) indicated no multicollinearity concerns. Sensitivity analyses excluding e-cigarette users and restricting the sample to participants who smoked ≥ 10 days in the past month produced substantively similar findings, underscoring the robustness of the results.

Overall, the results indicate that PME was positively associated with readiness to quit, both directly and indirectly through self-efficacy. Self-efficacy played a significant mediating role in this relationship. Nicotine dependence significantly moderated both the PME-self-efficacy and self-efficacy-readiness pathways, with stronger effects observed among youth with lower dependence. These findings support the proposed moderated mediation model and highlight the importance of both psychological and dependence-related factors in shaping quitting readiness.

## 4. Discussion

The present study provides novel insights into how PME influences readiness to quit among youth smokers in Guangdong province, emphasizing the mediating role of self-efficacy and the moderating impact of nicotine dependence. The findings extend prior work on health communication and smoking cessation by clarifying the psychological mechanisms through which anti-smoking messages exert their effects. Importantly, they suggest that youth smokers are a heterogeneous group in which dependence levels critically shape receptivity to persuasive health communication.

Our results align with social cognitive theory,^[15]^ highlighting self-efficacy as a central determinant of health behavior change. The partial mediation of PME by self-efficacy indicates that persuasive messages can strengthen agency beliefs, thereby facilitating readiness to quit. This supports previous findings that self-efficacy predicts both smoking reduction and long-term abstinence.^[5,10]^ Furthermore, the moderated mediation results underscore the complexity of behavioral change. High nicotine dependence appeared to constrain the translation of PME into self-efficacy, which consequently weakened readiness to quit. In line with Protection Motivation Theory, this reflects limitations in coping appraisals under high-dependence conditions.

These results also complement meta-analytic evidence showing the predictive validity of PME across health domains.^[3]^ Our findings expand this literature by demonstrating that PME operates within a psychological context influenced by addiction severity. This suggests that dependence should be considered when assessing message impact, adding nuance to communication theories that often treat message effects as uniform.

Self-efficacy emerged as a robust psychological mediator beyond message exposure alone. This finding is consistent with global health behavior research,^[16,17]^ and with Chinese studies showing that dependence interacts with cognitive beliefs to reduce cessation motivation.^[6,18]^ Our results extend this work by identifying nicotine dependence as a moderator of message-driven processes, further confirming its role as a psychological barrier to quitting.

Cross-cultural comparisons also suggest important nuances. In Western studies, message framing (gain vs loss) often determines PME.^[19]^ While framing remains important, our data indicate that in Chinese youth, the conversion of perceived persuasiveness into self-efficacy and intention is particularly critical: possibly reflecting the influence of collectivist values and social norms on health motivation.

From a practical standpoint, these findings have implications for tailoring communication strategies. For youth with low nicotine dependence, PME-enhancing campaigns may effectively increase self-efficacy and readiness. For those with higher dependence, however, PME alone may be insufficient; integrating persuasive messages with pharmacological aids and behavioral counseling is likely to yield stronger outcomes.^[20]^ Similarly, school- and college-based interventions should combine message-based approaches with peer-led support, given the strong social influence on adolescent smoking.^[21]^ At the policy level, sustained mass media campaigns and stricter advertising regulations remain essential, consistent with World Health Organization recommendations.^[7]^

The study possesses several strengths, including validated measures, robust moderated mediation analyses, and sensitivity checks. Nevertheless, limitations should be acknowledged. First, the cross-sectional design limits causal inference; longitudinal research could clarify whether PME-induced increases in self-efficacy lead to actual cessation. Second, self-reporting introduces potential bias, which future work could address using objective biomarkers such as cotinine levels. Third, the sample was drawn from Guangdong Province, and generalization to other regions should be made with caution.

Building on these findings, future research should test whether repeated message exposure sustains or amplifies self-efficacy over time. Experimental work could identify which message features, such as narrative versus factual appeals, most effectively enhance efficacy in Chinese youth. Additionally, studies exploring digital health tools (e.g., social media campaigns, mobile health apps) may identify scalable strategies for delivering persuasive and efficacy-enhancing messages. Integrating psychosocial communication with structural interventions, such as smoke-free campus policies and taxation measures, may ultimately yield synergistic effects on youth cessation outcomes.

## 5. Conclusion

This study demonstrated that PME plays a pivotal role in shaping quitting readiness among youth smokers in Guangdong, with self-efficacy acting as a partial mediator. At the same time, nicotine dependence attenuated both the impact of PME on self-efficacy and the influence of self-efficacy on readiness to quit, underscoring the importance of considering addiction severity when evaluating the impact of anti-smoking communication.

These findings highlight that persuasive health messages should aim not only to enhance perceived persuasiveness but also to strengthen self-efficacy, particularly among young smokers. For individuals with higher nicotine dependence, integrating supportive interventions, such as behavioral counseling or pharmacological aids, may be necessary to achieve meaningful cessation outcomes. Future research should employ longitudinal and experimental designs to confirm these mechanisms and to develop culturally tailored communication strategies that further improve the effectiveness of tobacco control efforts in China.

## Acknowledgments

No external funding was received for this study, and the authors declare no conflicts of interest. The authors would like to express their sincere gratitude to the participants who took part in this study, as well as to the institutions that facilitated data collection. Special thanks are extended to the journal editors and anonymous reviewers for their valuable feedback and constructive suggestions, which greatly improved the quality of this manuscript.

## Author contributions

**Investigation:** Yang Zhao.

**Methodology:** Xuelan Wu, Xiaoyu Tao.

**Supervision:** Xiaoyu Tao.

**Writing – original draft:** Yang Zhao.

**Writing – review & editing:** Xuelan Wu, Xiaoyu Tao.
